# Alternative Splicing in the Heart: The Therapeutic Potential of Regulating the Regulators

**DOI:** 10.3390/ijms252313023

**Published:** 2024-12-04

**Authors:** Francesca Briganti, Zilu Wang

**Affiliations:** 1 Division of Genetics, Department of Pediatrics, University of California San Diego, La Jolla, CA 92093, USA; 2 Division of Cardiology, Department of Pediatrics, University of California San Diego, La Jolla, CA 92093, USA

**Keywords:** splicing, cardiac differentiation, RBM20

## Abstract

Alternative splicing allows a single gene to produce a variety of protein isoforms. Changes in splicing isoform usage characterize virtually every stage of the differentiation process and define the physiological differences between cardiomyocytes with different function, at different stages of development, and pathological function. Recent identification of cardiac splicing factors provided insights into the mechanisms underlying alternative splicing and revealed how these splicing factors impact functional properties of the heart. Alterations of the splicing of sarcomeric genes, cell signaling proteins, and ion channels have been associated with the development of pathological conditions such as cardiomyopathy and arrhythmia. RBM20, RBM24, PTBP1, RBFOX, and QKI play key roles in cardiac development and pathology. A better understanding of their regulation will yield insights into healthy cardiac development and inform the development of molecular therapeutics.

## 1. Introduction

One gene can lead to the production of many different RNA isoforms via mechanisms such as alternative promoter usage, alternative splicing, and alternative polyadenylation. The RNA isoforms may differ in their coding potential, or in their regulatory regions, or in a combination of the two. Recent studies have shown extensive usage of alternative isoforms in various cell types [1,2,3,4], at different stages of development [5,6,7,8], and during pathogenesis [9,10,11,12]. This phenomenon is often referred to as transcript heterogeneity. It allows a relatively small number of genes to encode a wide variety of functions. Transcript heterogeneity, along with the combinatorial function of transcription factors, is one of the mechanisms proposed to resolve the G-value paradox, the apparent disconnect between an organism’s biological complexity and its number of protein coding genes [13,14]. Recent studies suggest that over 200,000 RNA isoforms are produced from the human genome [15] and that at least 75% of these have protein coding potential [16]. The ensemble of RNA isoforms generated from one gene and the pattern of regulatory signals leading to the generation of various RNA isoforms is known as the transcriptional landscape [17]. Alternative promoter/transcription start sites, alternative splicing, and alternative polyadenylation contribute to transcript heterogeneity. These mechanisms affect both the untranslated regions (UTRs), that control the regulation of the expression of the protein, and the open reading frame (ORF), that contains the information to make the protein. Alternative splicing allows a single gene to produce a variety of proteins. It is the co-transcriptional process in which the removal of various intronic regions from a pre-mRNA results in a variety of mature mRNAs encoding distinct proteins. The proteins produced from an alternatively spliced gene may have different to opposite roles [18]. Alternative splicing is a key contributor to transcript heterogeneity and plays a crucial role in defining cell identity through tissue-specific splicing modulators that coordinate the regulation of sets of exons. In tissue-specific splicing, subsets of alternatively spliced exons are subject to tightly regulated switches activating inclusion and/or exclusion in spatio-temporally regulated patterns. Many human diseases are caused by splicing defects, including spinal muscular atrophy, tauopathies, and Hutchinson-Gilford progeria syndrome [19]. The recent identification of several cardiac splicing factors, such as the RNA-binding motif protein 20 (RBM20) [20] and Quaking (QKI) [21], provided important insights into the mechanisms underlying alternative splicing and revealed how these splicing factors affect functional properties of the heart. Alterations in sarcomeric gene splicing, cell signaling proteins, and ion channels have been associated with the development of pathological conditions such as cardiomyopathy and arrhythmia.

## 2. Alternative Splicing

**Figure 1:**
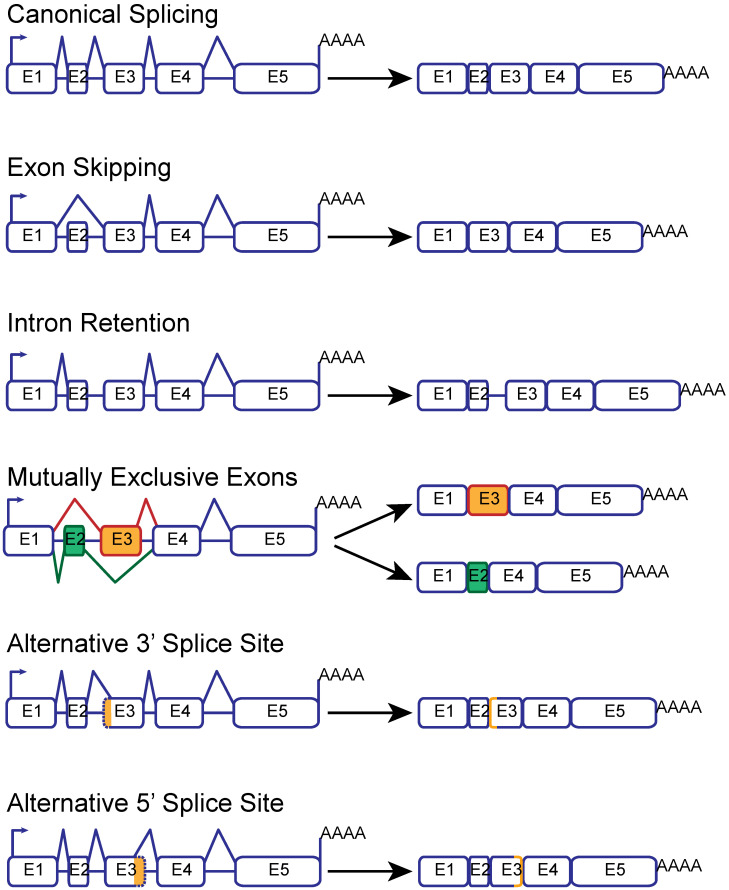
Schematic representation of the different types of alternative splicing and their effect on the mature mRNA product.

Splicing is the co-transcriptional process that leads to the removal of intronic regions from a pre-mRNA to form the mature mRNA (Figure 1, top). The molecular machinery responsible for this process is the spliceosome, a group of small nuclear ribonucleoproteins (snRNPs) in which the catalytic activity resides on the RNA portion. Most genes in eukaryotes undergo alternative splicing to produce multiple isoforms (Figure 1). The resulting proteins can have distinct activities or cell-type specificity. Alternative splicing is tightly controlled in different tissues at different developmental stages. The deregulation of splicing is associated with several human pathologies.

**Figure 2:**
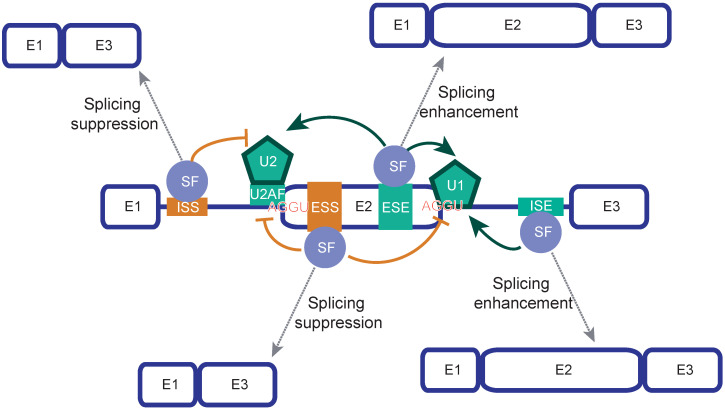
Schematic representation of the effect of exonic and intronic splicing regulatory regions. Spliceosome components and consensus sequences are also represented. E stands for exon; ESE is Exonic Splicing Enhancer; ESS is Exonic Splicing Silencer; ISE is Intronic Splicing Enhancer; ISS is Intronic Splicing Silencer: SF is Splicing Factor. Due to increasing evidence that splicing factors can have both enhancing and promoting effect on splicing, we use the nomenclature splicing factor to describe both inhibitors and activators of splicing. Splicing suppression results in exclusion of the regulated exon, splicing enhancement results in inclusion of the regulated exon.

Human introns are, on average, 5 kilobases long but can be as long as 250 kilobases. Introns are removed by cleavage at nearly invariant and unusually short consensus sequences called splice sites, usually GU at the 5’ end and AG at the 3’ end. The mechanics of splicing and alternative splicing are relatively well understood and have been extensively reviewed [22,23,24,25]. Given the size and relatively low complexity of splice site consensus sequences, it is not surprising that numerous sequences with a significant degree of consensus matching to authentic sites occur in most introns to form decoy splice sites. Two decoy splice sites within reasonable distance form pseudo-exons. While apparently indistinguishable from real exons, pseudo-exons are nearly never spliced in the mature mRNA. Pseudo exons are extremely common, yet the co-transcriptional process of cutting and pasting the pre-mRNA into a mature mRNA is extremely accurate, pointing to a role for additional sequence elements. In fact, numerous cis-acting regulatory RNA elements contribute to the process known as exon definition. They are collectively called Splicing Regulatory Elements (SREs). They are divided into intronic and exonic on the basis of their location and divided into enhancer and silencer on the basis of their activity. This produces all possible combinations: Exonic Splicing Enhancers (ESEs), Exonic Splicing Silencer (ESSs), Intronic Splicing Enhancers (ISEs), and Intronic Splicing Silencer (ISSs) (Table 1 & Figure 2). SREs in general function by recruiting trans-acting splicing factors that are responsible for the enhancement or suppression of different steps of the splicing reaction.

The majority of splicing factors described so far are involved in the regulation of the exon definition process and participate in the subsequent transition to the intron-spanning complex A, thus regulating the earliest steps of splicing [26]. The availability of different trans-acting splicing regulators in different cell types and at different cell states will determine the final outcome on regulated exons.

A well-studied example is the Polypyrimidine tract-binding protein (PTB), which typically inhibits splicing by binding to short poly-pyrimidine sequences. When binding to ESS, PTB can cause exon-skipping by different mechanisms: preventing the assembly of the exon definition complex, impeding the transition into an intron definition complex, or by binding directly to U1 snRNP and thus keeping it from engaging in interactions with other spliceosomal components [27]. Further complexity arises from the varying effects of splicing factors on the core spliceosomal components as a function of their position on the pre-mRNA. For example, oligo-G tracts can either promote splicing when located in the intronic region by recruiting heterogeneous nuclear ribonucleoprotein (hnRNP) H [28], or suppress splicing when located in exons, possibly by preventing the formation of the exon definition complex [29].

### The Role of Alternative Splicing in Regulating Cell Identity

The alternative proteins produced from an alternatively spliced gene may have different to opposite roles. One extreme example is Forkhead box P1 (FOXP1). An alternative splicing regulatory switch in FOXP1 regulates pluripotency and reprogramming [18]. In mouse Embryonic stem cells (ESCs), the pluripotency transcription factor Sal-like protein 4 (Sall4) has alternative splicing isoforms with different binding site preferences. The alternative splicing isoforms of Sall4 binding sites have partially different sequences, distinct epigenetic marks, and differential occupancy of other pluripotency factors [30]. This suggests that the different splice isoforms may differ in their regulatory output. Due to the complexity of splicing regulation, the function of most splicing regulatory proteins is difficult to predict, except when mutations in their genes cause disease.

## 3. Cardiac Development Regulation and the Emerging Role of Alternative Splicing Control

The heart is the first functioning organ in the developing human embryo. Cardiomyocyte specification begins during gastrulation and a heartbeat can be detected by ultrasound as early as 3.5 weeks after fertilization [31]. The heart is a complex organ with functionally and structurally distinct parts and a variety of cell types with diverse physiology. Both exposure to morphogenes and crosstalk between transcription factors are essential to control every step of heart development [32]. Cardiomyocyte progenitors are formed in the epiblast and travel through the primitive streak. These cells are characterized by the expression of the transcription factor Mesoderm posterior 1 (Mesp1). Mesp1 is activated by Brachyury (T-box transcription factor T), a mesoderm marker. Mesp1 promotes cell migration through the primitive streak and silences the pluripotency maintenance genes [33]. Both the location and the timing at which the cardiac progenitors travel through the primitive streak contribute to determining their fate, by exposing them to different signals. The cells passing through the streak closest to the primitive node will form the outflow tract, the cells passing through the mid-streak will form the ventricles, and the cells that will form the atria cross the streak most posteriorly. From a temporal point of view, the first cardiac progenitors to leave the primitive streak form an area of the lateral mesoderm called the cardiopharyngeal field from which the first heart field is formed. The first heart field will form the left ventricle and most of the atria, while a developmentally distinct group of cells known as the second heart field will sustain most of the right ventricle and the outflow tract development. From a gene regulation point of view, for cells in the first heart field, exposure to Bone morphogenetic protein 2 (BMP2) will turn on the expression of the cardiac transcription factors Nk2 homeobox 5 (NKX2-5), T-box transcription factor 5 (TBX5), and GATA binding protein 4 (GATA4). Cells in the second heart field are instead characterized by expression of a different transcription factor, ISL LIM homeobox 1 (ISL1). These and other transcription factors play a pivotal role in the commitment and development of cardiomyocytes. This is a relatively well understood process that has been described extensively [34]. On the other hand, the role of alternative splicing regulation has only began to emerge more recently and is often underappreciated.

### 3.1. Transcript Isoforms Complexity in Cardiac Development and Function

Some of the longest and most complex genes encoded by our genome are expressed in muscle cells. Different isoforms of the same gene are expressed in different types of muscle cells and contribute to the mechanical and electro-physiological differences that define muscle cell identity and specific function. Titin (TTN) is a representative example. TTN is the largest protein encoded by our genome [35]. Its gene includes nearly 400 exons and over 10 protein-coding splice-isoforms are currently annotated in Ensembl [36]. TTN is a structural component of the sarcomere. It is a spring-like protein that spans the length of the sarcomere and ensures its structural integrity. Different TTN isoforms confer different stiffness to the sarcomeres and the muscle cells [37,38,39]. TTN splicing is further complicated by the repetitiveness of its sequence and the necessity to coordinate the regulation of multiple exons at once [40,41]. Not only are different TTN isoforms expressed in different types of muscle cells [42] and in different types of cardiomyocytes [43], isoform composition is developmentally regulated in the heart to respond to the changing physiological needs of the developing organism [44]. Multiple splicing regulatory proteins have been implicated in the control of TTN slicing, including RBM20, RNA binding motif protein 24 (RBM24), and Sam68-like mammalian protein 2 (SLM2) [45,46,47]. Another sarcomeric gene that undergoes differential splicing during development is Troponin T2 (TNNT2). In the embryo and fetus, the full-length TNNT2 is expressed. However, exon 5 is excluded from the mature mRNA in adult cardiomyocytes [48].

Perhaps the first systematic study of alternative splicing in the developing heart came from Kalsotra et al. in 2008 [6]. In this seminal work, the authors used microarrays to identify alternative splicing events in embryonic and adult heart samples. They identified and validated 147 developmental switches in splicing patterns. Sequence analysis showed an enrichment for CUGBP and ETR-3-like factors (CELF), Muscleblind-like (MBNL), and Fox binding motifs. They identified CUGBP1 and MBNL1 as reciprocally expressed, with CUGBP1 highly expressed in the embryo and down-regulated in the adult heart and MBNL1 showing the opposite pattern. They used knockout (KO) mouse models to show that the reciprocal expression of these two splicing factors controlled about half of the alternative splicing events in the embryonic vs. adult heart. In the past two decades, numerous studies have examined a wide range of cardiac splicing factors that regulate cardiac development and various cardiac physiological functions (Table 2).

### 3.2. RBM20

**Figure 3:**
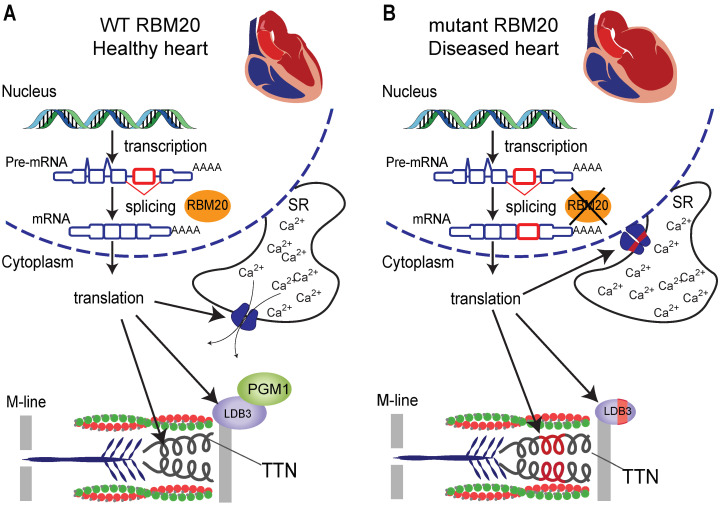
Schematic of RBM20 effect on splicing and heart health. (**A**) WT RBM20 promotes skipping of target exons and favors the production of heart specific isoforms of important cardiac genes, including TTN, RyR2 and LDB3. (**B**) Mutant RBM20 fails to suppress splicing of target exons and leads to loss of strength and dilation, hallmarks of DCM.

RBM20 expression increases gradually during embryonic development. As RBM20 expression increases, TTN splicing patterns change; shorter, stiffer isoforms become prevalent [49]. RBM20 acts as a splicing suppressor and induces skipping of target exons. Although TTN is one of the most studied and best understood targets of RBM20, other targets are similarly interesting (Figure 3, left panel). LIM domain binding 3 (LDB3) is a Z-line protein and the isoform favored by RBM20 is capable of interacting with phosphoglucomutase 1 (PGM1). It has been speculated that the function of this interaction is to coordinate mitochondrial efforts with sarcomeric needs [45,50]. A small exon in the Ryanodine receptor 2 (RyR2) is also regulated by RBM20 [51]. Splicing of this exon is predicted to regulate the subcellular localization of RyR2 and therefore affect calcium handling [52].

### 3.3. RBM24

RBM24 is expressed in skeletal and cardiac muscle and plays a key role in defining muscle identity. Its role in cardiogenesis was initially studied in zebrafish. RBM24 knock-out zebrafish embryos displayed reduced sarcomeric proteins expression, sarcomere abnormalities, loss of contractile force in cardiomyocytes, and deficient circulation [53,54]. Similarly, RBM24 depletion in human embryonic stem cells followed by induction of cardiac differentiation resulted in defective splicing of key myofibrillar components such as TTN, Actinin alpha 2 (ACTN2), and Myosin X (MYO10); defective contractility; and consequent failure to complete myofibrillogenesis [55]. RBM24 also controls the alternative splicing of pluripotency factors, and therefore its absence has been linked to the inability of pluripotent stem cells to exit the pluripotency state as necessary to undergo differentiation [56,57].

### 3.4. PTBP1

Polypyrimidine tract-binding protein 1 (PTBP1) is a pleiotropic RNA binding protein capable of regulating mRNAs at multiple levels: splicing, stability, and translation. PTBP1 is widely expressed in different cell types and contributes to neuronal as well as muscle differentiation [58,59,60]. PTBP1 is highly expressed in the embryonic myocardium and it is quickly downregulated after birth allowing the adult isoforms of target genes to be expressed. PTBP1 targets include Tropomyosin 1 and 2, Myocyte enhancer factors 2A and 2B (Mef2a and Mef2b, respectively), TNNT2, and ACTN [61,62,63].

### 3.5. RBFOX

Both RNA binding Fox-1 homologs (RBFOX), RBFOX1 and RBFOX2, play key roles in heart development. However, differently from previously described splicing regulators, RBFOX seems to have an earlier role in heart development and appears to regulate earlier events such as cell adhesion and intercellular communication [64,65]. RBFOX variants have been linked to congenital heart defects [66]. A recent study in zebrafish showed that RBFOX is required for proper splicing of mitochondrial, sarcomeric, and cytoskeletal genes [67]. RBFOX2 also plays a vital role in early cardiac development, regulating genes such as TNNT2 and RYR2 [65]. RBFOX2 primarily targets genes involved in sarcomere assembly, ion channels, and contractility, including calcium voltage-gated channel subunit alpha1 C (CACNA1C) and Ankyrin 2 (ANK2), which encodes a protein that anchors membrane proteins to the cytoskeleton. Mutations or dysregulation of RBFOX2 are associated with congenital heart defects [65,68] as well as other pathologies. RBFOX2 is dysregulated in diabetic hearts [69] and in a study of parent-offspring trios with congenital heart disease (CHD), RBFOX2 was one of the genes identified as contributing to the extracardiac congenital anomalies and neurodevelopmental disabilities [70].

### 3.6. QKI

QKI is ubiquitously expressed and mostly known for its role in the brain and in neurodegeneration [71,72,73,74]. A role for QKI in cardiovascular development and function is only starting to emerge. In 2016 single nucleotide polymorphisms (SNPs)near QKI’s locus were associated with congenital heart defect [75]. A recent study found that QKI is highly expressed in developing and adult cardiomyocytes [76]. More recent studies have shown that QKI is essential for muscle-specific splicing regulation and therefore for cardiac function [21,77].

**Table 2:** Summary of splicing factors that control heart development and physiology with their gene and mechanisms targets.

Splicing Factor	Regulated Gene Targets	Regulated Mechanisms	Evidences
RBM20	LDB3, RYR2, TTN	Sarcomere structure; E-C coupling	In vitro [45,49,50,51,52]; Mouse [49]; Rat [45,50]; Human [45,49];
RBM24	ACTN2, MYO10, TTN	Myofibrillogenesis; contractility; differentiation	In vitro [53,55,56,57]; Mouse [57]; Zebrafish [53,54]
PTBP1	ACTN, MEF2A, MEF2B, TNNT2, TPM1, TPM2	Cardiac development	In vitro [59,61,62,63]; Mouse [59,61]; Rat [59]
RBFOX1	MEF2; huG, actn3a, ptpla, camk2g1, ktn1 ^1^	Cardiac development; contractility	Mouse [78]; Zebrafish [64]; Human [78]
RBFOX2	Abi1, Ect2, Fn1 ^2^	Cardiac development; cell-ECM adhesion and signaling; cell cycle progression	In vitro [65,69]; Mouse [65,69]; Zebrafish [66]; Human [68,69,70]
QKI	ACTN2, CAMK2D, RYR2	Myofibrillogenesis; calcium dynamics; E-C coupling	In vitro [21,77]; Mouse [21,77]; Human [75]

^1^ Gene targets in zebrafish model. ^2^ Gene targets in mouse model.

## 4. Aberrant Splicing in Cardiac Disease

Tissue-specific alternative splicing has been difficult to study due to the combinatorial nature of alternative splicing both at the level of exon inclusion/exclusion and at the level of splicing regulators synergistic/antagonistic effect. Disease-causing mutations that alter splicing in a tissue-specific manner offer an opportunity to investigate tissue-specific alternative splicing (Table 3).

For the heart, RBM20 is a relatively well studied example. Mutations in its gene cause a severe form of dilated cardiomyopathy (DCM) along with aberrant splicing of over 100 exons in at least 40 genes. One of the best understood functions of RBM20 is the regulation of sarcomere stiffness through the splicing of TTN. In DCM, a deficient RBM20 causes the production of a longer, more compliant TTN (Figure 3, right panel). Increased compliance of the sarcomere aligns well with our understanding of DCM in which the walls of the heart lose passive stiffness with consequent increased chamber size (especially of the left ventricle) and systolic dysfunction [79]. Low expression of RBM20 in the absence of coding sequence mutations has also been associated with splicing defects similar to those observed in the presence of *RBM20* mutations [45]. These and other studies support the idea that mutations in *RBM20* cause loss of function, at least in its splicing regulatory role. Recent studies, however, have uncovered unexpected cytoplasmic functions for some RBM20 mutants, suggesting a gain of function effect [80,81]. RBM20-dependent splicing has also been linked to restrictive cardiomyopathy (RCM). While more rare than DCM, RCM is the deadliest form of pediatric heart disease. It is characterized by abnormally rigid walls of the ventricles that lack the flexibility to expand as the ventricles fill with blood. Over time, the heart loses the ability to pump blood properly leading to heart failure (HF) (diastolic dysfunction), arrhythmia, and sudden death. Therapeutic options are limited, and many patients go on to require pediatric heart transplantation [82,83]. Although the molecular mechanism for excessive rigidity in the ventricles in RCM is not fully understood, it is known that intrinsic stiffness of the heart wall is determined in large part by the compliance of TTN. Indeed, insufficient TTN is thought to be an important contributor to the development, progression and pathophysiology of RCM [84]. Prior work has demonstrated that inhibition of RBM20, either through genetic mutations or pharmacologically, improves TTN compliance and can ameliorate diastolic dysfunction in mice [85,86]. The co-occurrence of mutations in RBM20 and one of its most notable targets, TTN, was described in a case of severe and early onset DCM. Family members of the index patient with only one of the mutations have normal splicing of TTN and RYR2 and a milder cardiac phenotype, suggesting that aberrant splicing results from a compounding effect when both the splicing regulator and the target gene are mutated [87].

Multiple lines of evidence suggest that, through various mechanisms, RBM24 plays a protective role in the heart under disease conditions. These include promoting fibrosis, inhibiting apoptosis via p53 modulation, and preventing cardiac hypertrophy in cooperation with RBM20 [88,89,90]. In adult mouse cardiomyocytes, overexpression of RBM24 induces fibrosis through upregulation of fibrosis-associated genes, particularly within the Transforming growth factor beta (TGF-β) signaling pathway. Typically, TGF-β activates fibroblasts and promotes the deposition of extracellular matrix components, which may help to reinforce injured heart tissue. However, it is shown that TGF-β signaling in cardiomyocytes also contributes to fibrotic response. It remains to be investigated whether differential expression of RBM24 promotes fibrosis directly through regulating factors in the TGF-β signaling pathway or indirectly through regulating immune response mechanisms such as inflammatory cytokines [88]. In addition to fibrosis, RBM24 regulates apoptosis through its effect on p53, a transcription factor that controls cell cycle arrest and apoptosis in response to cellular stress. By binding to p53 mRNA and inhibiting its translation, RBM24 prevents excessive apoptosis in cardiac cells. It is worth noting that the expression of RBM24 inversely regulates the translation of p53 in both directions. The balance between these two factors is crucial for preserving cardiac cell populations and preventing congenital cardiac defects during embryonic development [89]. As a splicing regulator, RBM24’s protective role in cardiomyopathy is further supported by its regulation of the alternative splicing of PDZ-LIM proteins (ENH). ENH1 is co-regulated by RBM20 and RBM24, and alternatively spliced towards short isoforms (ENH3 and ENH4) that lack LIM domains. While the ENH1 isoform promotes hypertrophic signaling complexes and leads to pathological cardiac hypertrophy when disregulated, these shorter, LIM-less ENH3 and ENH4 isoforms inhibit hypertrophic remodeling, counteracting the effects of the full-length isoform. By shifting ENH splicing towards short, cardioprotective isoforms, RBM20 and RBM24 collectively prevent hypertrophic remodeling in the heart [90].

Another example of the complexity of alternative splicing regulation in the diseased heart is exemplified by PTBP1 dysregulation in diabetic cardiomyopahty, where a splicing regulator can also be the target of a different splicing regulator [91]. Typically, PTBPs are splicing repressors that contribute to the developmental process of various tissues. In Type-1 diabetic heart, PTBP1 splicing is altered to favor a developmental isoform over the adult one. It is suggested that this splicing switch is co-modulated by CELF1 and RBFOX2, both of which are dysregulated in diabetic hearts. In turn PTBP1 and RBFOX2 compete for the same splice targets and their relative abundance, in conjunction with that of CELF1, determine the prevailing isoforms. As a crucial splicing regulator in cardiac development, PTBP1 also plays a key role in ventricular chamber morphogenesis [92]. Enriched in cardiac endothelial cells during development, PTBP1 modulates the splicing of Arrestin beta 1 (ARRB1), an adapter protein associated with G protein-coupled receptor (GPCR) signaling that is also shown to be necessary for the migration of cardiac endothelial cells. In embryonic mouse heart, depletion of PTBP1 in endothelial cells alters the splicing of ARRB1 to a longer isoform, reducing endothelial cell migration, disrupting cardiomyocyte proliferation, and eventually leading to a left ventricular noncompaction phenotype.

When comparing transcriptome-wide alternative splicing events between healthy and failing hearts, RBFOX1 comes out as one of the most differentially expressed splicing regulators. In healthy developing mouse and zebrafish hearts, expression of RBFOX1 is typically elevated during cardiac development. However, in overload-induced heart failure, it is significantly repressed at both transcriptional and translational levels [93]. As a splicing regulator, RBFOX1 directly modulates isoform switching of variants within the MEF2 transcription factor family. It is reported that the aberrant splicing of MEF2 mediated by diminished RBFOX1 contribute to hypertrophy and HF, while the reintroduction of RBFOX1 is able to rescue the diseased hearts [78]. Similarly, RBFOX2 is also recognized as a major player in cardiac diseases. RBFOX2 deficiency is observed in congenital heart defects such as hypoplastic left heart syndrome [68], disease development in diabetic hearts [69], and CHD that co-occurs with extracardiac congenital anomalies and neurodevelopmental disabilities [70].

QKI has been a well-studied target in many neurological disorders and recent studies reveal that its role as a splicing regulator is critical for cardiac development and function. QKI regulates alternative splicing events for genes that are closely associated with cardiac myofibrillogenesis [21]. In particular, absence of QKI alters normal splicing of ACTN2, which negatively impacts myofibril structure by disrupting Z-line formation. Additionally, phenotypic differences in calcium dynamics and E-C coupling are attributed to QKI-mediated alternative splicing events. Furthermore, it is also shown in adult cardiomyocytes that QKI is responsible for maintaining striated muscle identity by regulating muscle-specific alternative splicing [77].

### 4.1. Heart Failure and the Global Disruption of Physiological Splicing

Heart failure is a pathological condition in which the heart fails to provide the body with appropriate blood flow. Heart failure can have many distinct causes and many forms of heart disease eventually lead to heart failure. Heart failure is characterized by global changes in splicing patterns [94,95]. Recent studies suggest that global changes in splicing factors constitute a reactivation of fetal splicing factors due to a reversion to fetal splicing program [96] and overall changes in the composition of the spliceosome [97].

### 4.2. The Regulation of Cardiac Splicing Factors

Just as any other protein, splicing regulatory proteins can and are regulated at different levels of gene expression: transcriptional, post-transcriptional, and post-translational. It is commonly accepted that SR proteins are regulated by phosphorylation of the RS domain [98]. By changing the charges on the domain responsible for protein-protein interactions, phosphorylation of the RS domain controls both nuclear import [99] and interaction with other proteins that have RS domains [100,101]. Notably, DCM-causing mutations cluster in the RS domain of RBM20 [102] and some of these mutations have been shown to interfere with both the protein’s ability to interact with other splicing regulatory proteins [103] and nuclear shuttling [104]. While many studies have focused on the identification of RBM20 targets, few have looked at the kinases regulating RBM20 protein [105] and only indirect evidences exist about the transcriptional control of RBM20 expression [51]. While the study of RBM20 targets is important to understand how mutations in its gene cause DCM, RBM20 regulation is where the therapeutic efforts should be focused to counteract disease development at its root rather than at its effects. Even less is known about the regulation of RBM24, despite its potential cardioprotective role. PTBP1 is known to be regulated at the co-transcriptional level and its aberrant splicing has been linked to pathogenesis [91], yet the players responsible for this regulation remain unknown.

## 5. Recent and Future Work

Established technologies and bioinformatics tools enable researchers to study the targets of splicing regulatory proteins by RNAseq following gene knock-out or knock-down. These technologies require little customization: siRNA targeting any sequence can be purchased and used to produce a knockdown and RNAseq is agnostic to the treatment give to the samples [106]. However studying the protein’s regulation requires a more “custom” approach and has, thus far, proven more challenging. Despite its challenges, a deep understanding of splicing regulators’ complex regulation is of paramount importance when splicing of their targets becomes the objective of a therapeutic strategy as shown by an elegant study by Wilkins and colleagues recently published in Science [107]. Recently, we have developed a strategy to study regulators of RBM20 in the context of human cardiomyocytes by harnessing the potential of genome editing technologies, the ability of pluripotent stem cells to efficiently differentiate into cardiomyocytes, and high-throughput high content imaging [108].

## 6. Conclusions

The dysregulation of RNA binding proteins is emerging as a key driver of human disease. Researchers are turning their attention to the therapeutic potential of the regulation of RNA binding protein expression and activity [107,109]. A systematic understanding of RNA binding protein regulation in relevant cell types is a first necessary step to harness a new opportunity for treating cardiovascular disease.

## 7. Limitations

This review is not a comprehensive discussion of alternative splicing nor of cardiac function and dysfunction. Numerous high-quality sources are available for a deeper understanding of the mechanisms of alternative splicing, for the broader context of cardiac physiology, and for details on genetic cardiac disease. The purpose of this review is to highlight the importance of alternative splicing regulation in cardiac physiology and pathogenesis and to point out its therapeutic potentials. Other topics are touched upon to help readers with diverse background and expertise put the discussion into context.

## Figures and Tables

**Table 1:** Summary of SRE, their location, and their effect on splicing.

Type of SRE	Acronym	Sequence Location	Effect on Target Exon
Exonic Splicing Enhancer	ESE	Exon	Exon inclusion
Exonic Splicing Silencer	ESS	Exon	Exon skipping
Intronic Splicing Enhancer	ISE	Intron	Exon inclusion
Intronic Splicing Silencer	ISS	Intron	Exon skipping

**Table 3:** Summary of splicing factors role in cardiac disease.

Heart Condition	Splicing Factor
Dilated cardiomyopathy (DCM)	RBM20
Restrictive cardiomyopathy (RCM)	RBM20
Heart Failure (HF)	RBM20; RBFOX1; RBFOX2
Hypertrophic cardiomyopathy (HCM)	RBM20; RBM24; RBFOX1; RBFOX2
Cardiac fibrosis	RBM24
Congenital heart disease (CHD)	RBM24; RBFOX1; RBFOX2; QKI
Diabetic cardiomyopathy	PTBP1; RBFOX1; RBFOX2; QKI

## Data Availability

Not applicable.

## Abbreviations

The following abbreviations are used in this manuscript:

ACTN2	Actinin alpha 2
ANK2	Ankyrin 2
ARRB1	Arrestin beta 1
BMP2	Bone morphogenetic protein 2
CACNA1C	Calcium votage-gated channel subunit alpha1 C
CELF	CUGBP and ETR-3-like factor
CHD	Congenital heart disease
DCM	Dilated cardiomyopathy
ENH	PDZ-LIM protein
ESC	Embryonic stem cell
ESE	Exonic Splicing Enhancer
ESS	Exonic Splicing Silencer
FOXP1	Forkhead box P1
GATA4	GATA binding protein 4
GPCR	G protein-coupled receptor
HCM	Hypertrophic cardiomyopathy
HF	Heart failure
hnRNP	Heterogeneous nuclear ribonucleoprotein
ISE	Intronic Splicing Enhancer
ISL1	ISL LIM homeobox 1
ISS	Intronic Splicing Silencer
KO	Knockout
LDB3	LIM domain binding 3
MBNL	Muscleblind
MEF2	Myocyte enhance factor 2
Mesp1	Mesoderm posterior 1
MYO10	Myosin X
NKX2-5	Nk2 homeobox 5
ORF	Open reading frame
PGM1	Phosphoglucomutase 1
PTB	Polypyrimidine tract-binding
PTBP1	Polypyrimidine tract-binding protein 1
QKI	Quaking
RBFOX	RNA binding fox-1 homolog
RBM20	RNA-binding motif-20
RBM24	RNA-binding motif-24
RCM	Restrictive cardiomyopathy
RyR2	Ryanodine receptor 2
Sall4	Sal-like protein 4
SLM2	Sam68-like mammalian protein 2
SNP	Single nucleotide polymorphism
snRNP	small nuclear ribonucleoprotein
SRE	Splicing Regulatory Element
TBX5	T-box transcription factor 5
TGF-β	Transforming growth factor beta
TNNT2	Troponin T2
TTN	Titin
UTR	Untranslated region
